# Detecting Blood Methylation Signatures in Response to Childhood Cancer Radiotherapy via Machine Learning Methods

**DOI:** 10.3390/biology11040607

**Published:** 2022-04-15

**Authors:** Zhandong Li, Wei Guo, Shijian Ding, Kaiyan Feng, Lin Lu, Tao Huang, Yudong Cai

**Affiliations:** 1 College of Food Engineering, Jilin Engineering Normal University, Changchun 130052, China; lizd591@jlenu.edu.cn; 2 Key Laboratory of Stem Cell Biology, Shanghai Jiao Tong University School of Medicine (SJTUSM) & Shanghai Institutes for Biological Sciences (SIBS), Chinese Academy of Sciences (CAS), Shanghai 200025, China; gw_1992@alumni.sjtu.edu.cn; 3 School of Life Sciences, Shanghai University, Shanghai 200444, China; dingshijian@shu.edu.cn; 4 Department of Computer Science, Guangdong AIB Polytechnic College, Guangzhou 510507, China; kyfeng@gdaib.edu.cn; 5 Department of Radiology, Columbia University Medical Center, New York, NY 10032, USA; 6 Bio-Med Big Data Center, CAS Key Laboratory of Computational Biology, Shanghai Institute of Nutrition and Health, University of Chinese Academy of Sciences, Chinese Academy of Sciences, Shanghai 200031, China; 7 CAS Key Laboratory of Tissue Microenvironment and Tumor, Shanghai Institute of Nutrition and Health, University of Chinese Academy of Sciences, Chinese Academy of Sciences, Shanghai 200031, China

**Keywords:** methylation, childhood cancer radiotherapy, machine learning method, feature selection, rule learning

## Abstract

**Simple Summary:**

Radiotherapy for cancer patients can cause abnormal DNA methylation. We developed a computational workflow that can identify crucial methylation alterations related to treatment exposure in childhood cancer survivors.

**Abstract:**

Radiotherapy is a helpful treatment for cancer, but it can also potentially cause changes in many molecules, resulting in adverse effects. Among these changes, the occurrence of abnormal DNA methylation patterns has alarmed scientists. To explore the influence of region-specific radiotherapy on blood DNA methylation, we designed a computational workflow by using machine learning methods that can identify crucial methylation alterations related to treatment exposure. Irrelevant methylation features from the DNA methylation profiles of 2052 childhood cancer survivors were excluded via the Boruta method, and the remaining features were ranked using the minimum redundancy maximum relevance method to generate feature lists. These feature lists were then fed into the incremental feature selection method, which uses a combination of deep forest, k-nearest neighbor, random forest, and decision tree to find the most important methylation signatures and build the best classifiers and classification rules. Several methylation signatures and rules have been discovered and confirmed, allowing for a better understanding of methylation patterns in response to different treatment exposures.

## 1. Introduction

Radiotherapy (RT) has been an important and effective anticancer treatment for over a century. Approximately 70% of all patients with cancer are treated via RT alone or in combination with other treatment approaches [1]. The application of RT in cancer treatment has largely improved the short-term survival of patients [2]. High doses of radiation can kill cancer cells and shrink tumors as electrically charged particles pass through tumor cells. DNA double-strand breaks are the classical outcomes induced by RT that can effectively arrest cell growth and induce cell death in tumor cells [3]. However, RT itself can also cause DNA damage in normal tissues and result in long-term morbidity and mortality. Many underlying molecular alterations induced by RT can cause long-term adverse health outcomes. Patients with cancer who had received RT reportedly suffered from sterile inflammation, premature senescence, and cardiometabolic diseases during long-term outcomes [4]. Therefore, biomarkers that indicate the potential risk of long-term outcomes after RT exposure must be identified.

Various cancer treatments, such as drug treatment, RT, or the combination of anticancer treatments, can potentially impact the methylation status of the genome, subsequently leading to gene regulation alterations and aberrant phenotypes [5]. Unlike genetics, which is relatively static and exerts a direct effect on gene encoding, methylation modification is thought to be plastic and can be modified in response to environmental stimulations [6]. Radioactive exposure might cause the acquisition of soma-wide alterations in DNA methylation. Accumulating evidence supports the idea that DNA methylation abnormalities are closely associated with a diverse group of diseases [7]. The direct effects of radiation on DNA methylation had been reported as early as 1989 by Kalinich et al. [8]. They observed a decrease in 5-methylcytosine after γ radiation irradiation in cell lines in vitro. Pogribny et al. [9] also found different DNA methylation patterns at various doses of X-ray exposure. A hypothesis suggests that the alteration of DNA methylation may reflect biological responses to radiation that will lead to specific sensitivity to RT [10]. Although the association between DNA methylation alteration and health outcomes has been widely reported, the underlying biological mechanisms by which radioactive exposure affects methylation modifications are still incompletely understood. Moreover, the methylation patterns induced by region-specific RT require further research. Therefore, we focused on the key DNA methylation alterations associated with specific cancer RT and the functional role of such methylations in health outcomes.

In this study, we computationally analyzed the methylation profiles of patients who underwent RT. A recent publication obtained the methylation data from 2052 cancer survivors by using DNA methylation microarray [11]. On the basis of these public data, we conducted a machine learning analysis to identify the key methylation sites that may be relevant to RT exposure and its long-term adverse health outcomes. Furthermore, we divided this cohort into four categories according to the types of treatment exposures, namely, abdominal RT, brain RT, chest RT, and pelvic RT. Subsequently, we applied the minimum redundancy maximum relevance (mRMR) and incremental feature selection (IFS) methods to identify the most relevant methylation sites for predicting each type of RT, and then we constructed decision rules for the quantitative description of the relationship between the methylation sites and RT. Overall, our study sheds light on the potential methylation modifications in response to region-specific cancer RT.

## 2. Materials and Methods

### 2.1. Datasets

The methylation datasets of childhood cancer survivors were obtained from the Gene Expression Omnibus database with the accession number GSE169156 (https://www.ncbi.nlm.nih.gov/geo/query/acc.cgi?acc=GSE169156 (accessed on 6 April 2021)) [11]. The datasets included the blood DNA methylation profiles of patients who underwent abdominal RT, brain RT, chest RT, and pelvic RT. Table 1 shows the number of positive and negative samples for each RT. Each sample had the methylation levels of 865,892 sites measured with Illumina Infinium HumanMethylation850 BeadChip which included 866,895 probes. Some probes had too many missing values and were excluded from further analysis. The beta values of 865,892 methylation sites were analyzed. The beta values ranged from 0 to 1. A high beta value meant methylated while a low beta value meant un-methylated. All the data descriptions can be found at https://www.ncbi.nlm.nih.gov/geo/query/acc.cgi?acc=GSE169156 (accessed on 6 April 2021) and the annotation information of Illumina Infinium HumanMethylation850 BeadChip can be found at https://www.ncbi.nlm.nih.gov/geo/query/acc.cgi?acc=GPL23976 (accessed on 6 April 2021).

### 2.2. Boruta Feature Selection

Directly analyzing all methylation features in a dataset is difficult and time-consuming owing to the vast number of features in the dataset. In this study, we applied the Boruta feature selection method to remove irrelevant features [12]. The shadow features in Boruta were created by shuffling the original features, and then the feature matrix was created by connecting the original features and the shadow features were trained using random forest (RF). Finally, the feature importance score of each shadow feature was utilized as a reference to pick the feature set from the original features most closely related to the category variables. The methylation profiles were analyzed using the Boruta program (https://github.com/scikit-learn-contrib/boruta_py (accessed on 14 September 2020)), and the default parameters were adopted to run the program for convenience.

### 2.3. mRMR

mRMR is a powerful method in feature selection and has been widely applied in the field of biomedical research [13]. It evaluates the importance of features on the basis of mutual information (MI), which is defined as follows:

(1) $$I\left(X,Y\right)=\int \int p\left(x,y\right)log\frac{p\left(x,y\right)}{p\left(x\right)p\left(y\right)}dxdy,$$

where p(x,y) is the joint probability density function of *x* and *y*; and p(x) and p(y) are the marginal probability density functions of *x* and *y*, respectively. A high MI value indicates a great correlation between *X* and *Y*. Suppose *S* represents the set of features that have been selected, let us define the following optimization equations:

(2) $$\max D\left(S,c\right),\;D=\frac{1}{\left|S\right|}\sum_{x_{i}\in S}I(x_{i};c).$$

However, the features of *S* may have redundancies. The redundancy of set *S* is as follows:

(3) $$\min R\left(S\right),\;R=\frac{1}{{\left|S\right|}^{2}}\sum_{x_{i}x_{j}\in S}I\left(x_{i},x_{j}\right),$$

The objective of mRMR is to select the set *S* with the maximum relevance and the minimum redundancy, which is defined as follows:

(4) maxΦ(D,R), Φ=D−R.

Therefore, an increase in *D* and a decrease in *R* both contribute to an increase in the objective function. Given that there are already *m* − 1 features in *S*, then the *m*-th feature is selected from the remaining features to maximize Φ(D,R). In the end, mRMR outputs a ranked feature list of *m* features. In the present study, the mRMR program was obtained from http://www.home.penglab.com/proj/mRMR/ (accessed on 2 May 2018) and run with the default parameters.

### 2.4. IFS

IFS can determine the best number of features by using machine learning algorithms, such as RF, in a ranked feature list [14]. IFS can construct a succession of feature subsets on the basis of a given step interval *s* (i.e., 1) for a feature list *F* generated by mRMR. For example, the first feature subset $F_{1}$ contains the top 1×s features, whereas $F_{2}$ contains the top 2×s features, and so on. For each candidate feature subset $F_{i}$, a machine learning model will be trained on the samples comprising this feature subset. Using the 10-fold cross-validation [15] and synthetic minority oversampling technique (SMOTE) procedures, the evaluation metrics indicating the model’s performance, such as Matthews correlation coefficient (MCC), are obtained. Finally, the IFS curves are produced using the number of features as the x-coordinate and an evaluation metric as the y-coordinate. The highest point of the curves can be used to identify the best feature subset.

### 2.5. Classification Algorithms

In the IFS process step, four classification algorithms are used to build classifiers, namely, DF [16], kNN [17], RF [18], and DT [19], which are described in detail below. These algorithms have been widely used in tackling various medical problems [20,21,22,23,24,25,26,27,28,29,30].

#### 2.5.1. DF

DF combines numerous ensemble-based methods, such as RFs and stacking, to build a cascade structure that resembles a multilayer neural network, but each layer contains RFs instead of neurons. Each layer accepts the feature information processed by the previous layer and outputs the result to the next layer in this architecture. A multigranularity scanning method can increase the representation learning capabilities of the input with large dimensionality. The DF program was downloaded from https://github.com/LAMDA-NJU/Deep-Forest (accessed on 15 November 2020) and run with the default parameters.

#### 2.5.2. kNN

kNN is a basic supervised learning algorithm. The key idea of this algorithm is to calculate the distance (e.g., Euclidean distance) between a new instance and each training sample and then find the first k-nearest samples and determine the category of the new instance.

#### 2.5.3. RF

RF is an ensemble learning method that improves a model’s prediction ability by integrating a number of DTs. Each DT is trained using some randomly selected samples and features from the original dataset. For a test sample, RF integrates the decisions of each DT to arrive at the final decision by majority voting.

#### 2.5.4. DT

DT is a white-box model that gives interpretable decision rules, unlike the three machine learning methods discussed above. To divide occurrences and features, it creates a classification or a regression model on the basis of the IF-THEN structure. In this study, the Scikit-learn package was applied to execute kNN, RF, and DT by using the default parameters.

### 2.6. SMOTE

In this study, SMOTE was used to generate the sample data of minority classes because four methylation profiles are highly uneven [31]. According to the principle of kNN, this method calculates the distances between a sample and other samples in the minority class and then selects multiple samples, including the sample itself and some of its neighbors, to generate a new sample linearly. SMOTE was employed to balance the training set so that the number of samples from different classes was equal when evaluating the performance of a classification model with 10-fold cross-validation. The SMOTE program with default parameters from the imbalance-learn package was utilized for this analysis.

### 2.7. Performance Measurement

In the process of 10-fold cross-validation, classification accuracy (ACC), specificity (SP), sensitivity (SN), and MCC [32,33,34,35] were used as evaluation metrics. These metrics are calculated as follows:

(5) $$\mathrm{ACC}=\frac{TP+TN}{TP+FP+FN+TN},$$

(6) $$\mathrm{SP}=\frac{TN}{TN+FP},$$

(7) $$\mathrm{SN}=\frac{TP}{TP+FN},$$

(8) $$\mathrm{MCC}=\frac{TP\times TN-FP\times FN}{\sqrt{\left(TP+FP\right)\left(TP+FN\right)\left(TN+FP\right)\left(TN+FN\right)}},$$

where *TP*, *TN*, *FP*, and *FN* represent true positive, true negative, false positive, and false negative, respectively.

## 3. Results

**Figure 1:**
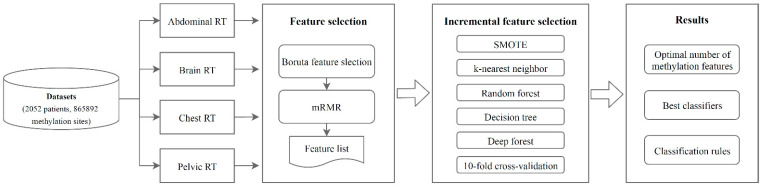
Computational workflow of this study. The methylation dataset was acquired in four sections from a public database: abdominal RT, brain RT, chest RT, and pelvic RT. The methylation features in each methylation profile were filtered and ranked using the Boruta feature selection and mRMR methods. The IFS method used the resulting feature list to identify the optimal number of features and develop the best classifiers and classification rules by combining SMOTE and classification algorithms.

In the present study, we proposed a computational workflow for analyzing the DNA methylation profiles of patients who underwent RT. Various feature selection methods and classification algorithms were used. Figure 1 depicts the entire study’s analysis flow, and the findings of this study are also presented.

### 3.1. Results of Feature Selection of the Methylation Datasets via the Boruta and mRMR Methods

Each original dataset contained 865,892 methylation sites, and the computational complexity of direct analysis was enormous. To address this issue, we initially employed the Boruta method to filter the features from the DNA methylation profiles of four tissues from patients who underwent RT. As a consequence, the number of retained features for abdominal RT, brain RT, chest RT, and pelvic RT was 766, 155, 972, and 257, respectively. The mRMR method was then utilized to construct four ranked feature lists according to the mRMR criterion, as shown in Table S1.

### 3.2. Results of IFS Method with Classification Algorithms

**Figure 2:**
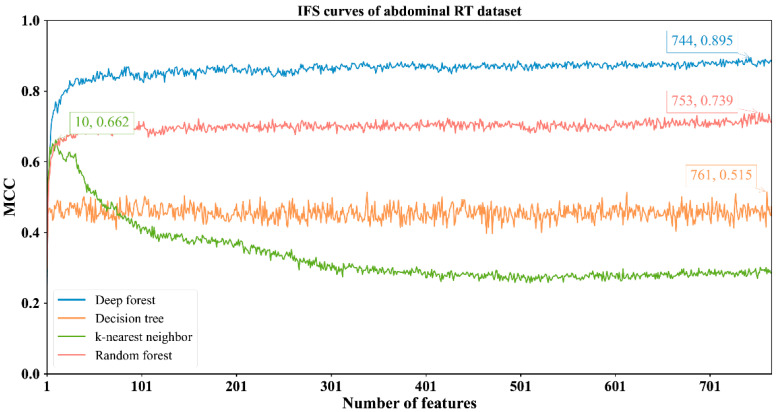
IFS curves with different classifiers on the different numbers of methylation features for abdominal RT methylation dataset. DF achieves the highest MCC value of 0.895 when the top 744 features are used.

**Figure 3:**
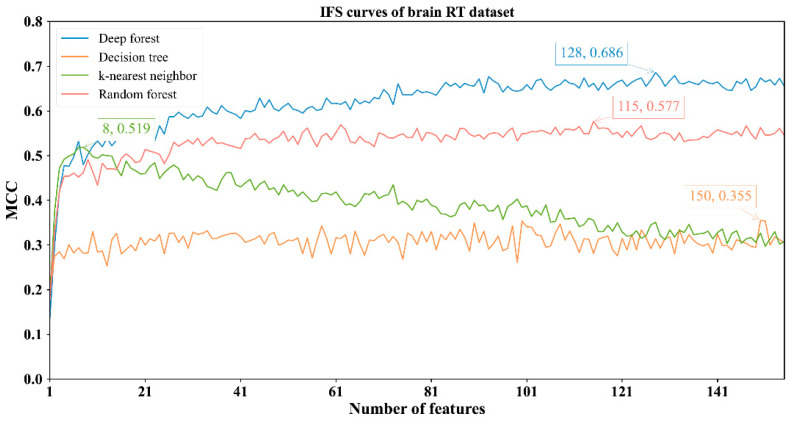
IFS curves with different classifiers on the different numbers of methylation features for brain RT methylation dataset. DF attains the highest MCC value of 0.686 when the top 128 features are utilized.

**Figure 4:**
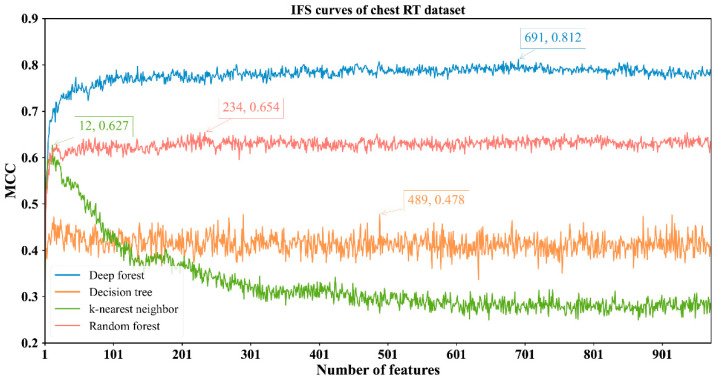
IFS curves with different classifiers on the different numbers of methylation features for chest RT methylation dataset. DF reaches the highest MCC value of 0.812 when the top 691 features are adopted.

**Figure 5:**
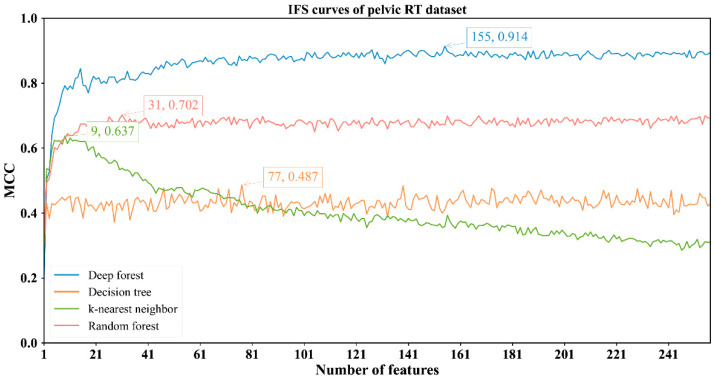
IFS curves with different classifiers on the different numbers of methylation features for pelvic RT methylation dataset. DF yields the highest MCC value of 0.914 when the top 155 features are employed.

The feature lists sorted by the mRMR method were fed into the IFS method with four classification algorithms to determine the best number of features. When the step size was set to 1, the first feature subset produced by IFS was the first feature in the list, the second feature subset was made up of the top two features, and so on. For example, the abdominal RT dataset yielded 766 feature subsets. Subsequently, four classification algorithms, namely, DF, kNN, RF, and DT, were adopted to build classifiers by using the sample data represented by these feature subsets and evaluation metrics were obtained. The performance of these classifiers with different feature subsets in different methylation datasets is provided in Tables S2–S5. The IFS curves were plotted with MCC as the vertical coordinate and the number of features as the horizontal coordinate, as shown in Figure 2, Figure 3, Figure 4 and Figure 5.

For the abdominal RT dataset, it can be observed from Figure 2 that four classification algorithms (DF, kNN, RF and DT) yielded the highest MCC of 0.895, 0.662, 0.739 and 0.515, respectively. These MCCs were obtained by using top 744, 10, 753, 761 features. Other measurements of these classification algorithms under corresponding features are listed in Table 2. Evidently, DF with the top 744 features provided the best performance. The MCC was at least 15% higher than the other highest MCCs yielded by the other three classification algorithms, suggesting the superiority of DF for identifying samples with abdominal RT.

For the other three datasets, DF still provided the highest MCC. In detail, DF with the top 128 features yielded the MCC of 0.686 on the brain RT dataset (Figure 3); DF with the top 691 features produced the MCC of 0.812 on the chest RT dataset (Figure 4); DF with the top 155 features generated the MCC of 0.914 on the Pelvic RT dataset (Figure 5). The ACC values of these DF classifiers were 0.869, 0.925, and 0.976 (Table 2), respectively. These values were all higher than those of the other three classification algorithms (Table 2). MCC was at least 10% higher and ACC was 5% higher, suggesting DF can capture more essential information in these datasets, thereby building more efficient classifiers.

From Figure 2, Figure 3, Figure 4 and Figure 5 and Table 2, some interesting phenomena can be observed. First, the performance of DF, kNN, RF and DT was uniform on four datasets. DF gave the best performance, followed by RF, kNN and DT. This result almost conformed to our general cognition. DF can be deemed as a generalized version of RF. Thus, it is generally more powerful than RF. kNN, in fact, is not a pure machine learning algorithm because it does not contain the training procedures. In most cases, it is weaker than RF. DT, as a rule-learning algorithm, cannot always provide high performance. Thus, its performance was the lowest in this study. However, its classification procedures are completely open, providing more clues to uncovering essential information behind the dataset. Second, the best kNN classifier adopted much fewer features than other three best classifiers on all datasets. kNN used about ten features to generate the highest MCC, whereas the other three algorithms need tens of, or even hundreds of, features to achieve the highest MCC. In the feature list yielded by the mRMR method, features with high ranks had a higher relationship with class labels. With a small number of top features in the list, kNN can easily distinguish positive and negative samples using a sample way (distance between samples). The other three algorithms adopted a much more complicated scheme to train the classifiers, these features were too few to build the optimum classifiers. However, when more and more features were added, more noises were included. As kNN does not contain the training procedure, it cannot identify interference information and exclude it, thereby influencing its performance. For the other three classification algorithms, their training procedures can help them extract useful information and build more powerful classifiers.

### 3.3. Classification Rules Extracted by the Optimal DT Classifiers

DF performed well in each methylation dataset. However, it is a black-box model that cannot provide quantitative rules. To extract the decision rules, we used the top 761, 150, 489, and 77 features from the abdominal RT, brain RT, chest RT, and pelvic RT datasets, respectively, to build the best DT classifiers. The expression rules obtained by the optimal DT classifier for each dataset are provided in Table S6. The abdominal RT, brain RT, chest RT, and pelvic RT datasets had 151, 239, 166, and 183 rules each. The number of rules for the positive and negative classes on each dataset is listed in Table 3.

## 4. Discussion

This study demonstrated that several optimal classifiers can recognize risk conditions, such as region-specific RT exposure, with relatively high ACC values on the basis of methylation profiles. In detail, we treated the methylation level of each site as the feature and identified the most relevant features through the Boruta and mRMR methods. The crucial DNA methylations that indicated abdominal RT, brain RT, chest RT, and pelvic-RT were individually estimated. We applied four different algorithms, namely, DF, kNN, RF, and DT, to construct the classifiers. DF was shown to have the best performance in the classification. Via feature selection, we identified 744 DNA methylation sites that were highly predictive of abdominal RT treatment. Moreover, we found that 128 crucial DNA methylation sites were associated with brain RT treatment. Furthermore, we determined that 691 DNA methylation sites were related to chest RT treatment. Finally, we recognized 155 key DNA methylation sites linked to pelvic RT treatment. We noticed that many methylation sites identified by our analysis had been reported to be significantly associated with RT treatment via epigenome-wide association study (EWAS) method by Song et al. [11], confirming the reliability of these feature selection methods. In detail, we compared the most relevant features related to each RT exposure by our analysis to the significant methylation sites (*p* < 9 × 10^−8^) from a previous EWAS study. Among the 330 methylation sites reported to be significantly associated with abdominal RT in the EWAS study, there were 169 methylation sites identified as highly related to abdominal RT in this study. The previous EWAS study reported nine methylation sites significantly associated with brain RT, and two of them were identified as highly predictive of brain RT by feature selection. Next, among 303 methylation sites significantly associated with chest RT exposure, there were 157 identified in the present study with the most relevance to chest RT. A total of 248 methylation sites were reported to be associated with pelvic RT by EWAS analysis. Of these, 113 methylation sites were identified in feature selection of pelvic RT exposure. Taken together, almost half of the previously reported methylation sites associated with RT treatment were identified again using a distinct computational method.

Essentially, the EMAS method is a set of statistical analysis approaches. In this study, we adopted quite different computational methods, i.e., machine learning algorithms, to reanalyze the blood DNA methylation profiles. These algorithms can deeply mine hidden relationships behind the datasets, including relationships between features and class labels or among features, which cannot be discovered by general statistical analysis approaches. Furthermore, the training procedures of such algorithms can help us improve the performance of classifiers. Thus, we obtained a different ranking of the feature’s relevance by comparison with the original EWAS study, showing an improved sensitivity and accuracy in identifying RT-related methylation modifications. The decision rules were built on the basis of the selected features, providing the criteria to indicate treatment exposures. To validate the relevance of these findings in distinguishing region-specific RT exposure, functional characteristics of these methylation sites were gathered from the literature, which supported a potential association between function injury and each type of treatment exposure. For each category of RT exposure, we presented detailed descriptions of the functional role of methylation modification related to RT.

### 4.1. Key Methylation Alteration Related to Abdominal RT

The CpG site cg21585138, which is located on chr3:5064516 and is mapped to the *CISH* gene, was identified as one of the most relevant features for indicating abdominal RT. *CISH* is involved in the IL-2 signaling pathway, and it is reportedly associated with infectious diseases [36]. The loss of *CISH* contributes to hyperproliferative responses in acute myelogenous leukemia [37]. Additionally, the methylation status of cg21585138 was found to be influenced by smoking, suggesting a potential epigenetic alteration caused by chemical toxicity [38]. This evidence supported the contention that cg21585138 may serve as a methylation signature for the risk of adverse health conditions. Abdominal RT allegedly exerts a harmful effect on health outcomes, and the methylation alteration at cg21585138 may be the early event after RT.

Another key CpG site, cg03054277, was identified to be highly predictive of abdominal RT. This methylation site is located on chr1:228400217 and mapped to the *OBSCN* gene. The protein product encoded by *OBSCN* is related to various functions, including transferase activity and tyrosine kinase activity. OBSCN reportedly plays a role in mediating cardiomyocyte adhesion via PI3K/AKT/mTOR signaling [39]. An epigenome-wide association analysis revealed that the methylation status of cg03054277 is associated with age, implying that it may be a senescence-related signature [40]. The CpG site cg03054277 is also identified as a DNA methylation biomarker of alcohol consumption [41]. Given that alcohol intake is viewed to cause the accumulation of body lesions, the methylation status of cg03054277 may indicate an initial signal for chemical toxicity. Therefore, cg03054277 may also act as the signature for abdominal RT.

Among the decision rules for identifying abdominal RT exposure, the CpG site cg17730048 was hypermethylated to indicate abdominal RT. This methylation site is located on chr17:26577563 within the CpG island region. Notably, cg17730048 is also identified as one of the risk signals associated with aging, suggesting that a high methylation level of cg17730048 may represent the impaired functional condition of an individual [40]. Moreover, this CpG site has been linked to maternal smoking in pregnancy [42]. This finding supported the idea that hypermethylation of cg17730048 may indicate the risk for adverse health conditions, consistent with our analysis that the hypermethylation of cg17730048 can predict abdominal RT.

### 4.2. Key Methylation Alteration Related to Brain RT

The most relevant CpG site for brain RT we identified was cg08866213, which is located on chr3:192530777 and mapped to the *MB21D2* gene. Overexpressed MB21D2 reportedly promotes a pro-oncogenic progression of head and neck cancer, and it also induces less sensitivity toward DNA-damaging agents, such as RT [43]. In addition, paclitaxel, a chemotherapy medication, allegedly results in the expression alteration of MB21D2 [44]. These results suggested that the *MB21D2* gene may be a key target in response to DNA-damaging agents, such as RT and chemotherapy. Therefore, we argue that changes in the methylation of cg08866213 can serve as a biomarker for indicating brain RT.

We also identified the CpG site cg15393490 as another important feature to indicate brain RT. This methylation site is located on chr1:207996459 and belongs to the promoter region of miR-29c. TCGA data indicated different methylation levels of cg15393490 in breast tumor subtypes [44]. Notably, miR-29c has been shown to be involved in many types of diseases, including ischemic brain injury [45]. We inferred that the methylation status of cg15393490 may be a risk indicator for brain injury, for example, the damage caused by brain RT. Furthermore, the DNA methylation of cg15393490 is reportedly associated with liver diseases and cholesterol metabolism [46,47]. These findings implied that the methylation status of cg15393490 is related to long-term adverse health conditions that may be caused by brain RT.

Among the rules identifying brain RT, we found that one CpG site, that is, cg18973101, was involved in several criteria. This CpG site is located on chr1:156251280 within the intergenic region between the *TMEM79* and *SMG5* genes. A high methylation level of cg18973101 is required to indicate brain RT. Several studies have discovered that the DNA methylation of cg18973101 is associated with long-term alcohol consumption [41,48]. The influence of alcohol consumption on the risk of disease is widely recognized, and DNA methylation may be one of the pathogenic mechanisms. We speculated that RT treatment may also exert a similar effect on DNA methylation alteration in cg18973101, making this key CpG site one of the signatures indicating brain RT exposure.

### 4.3. Key Methylation Alteration Related to Chest RT

We identified the CpG site cg01511232 as one of the most relevant features for predicting chest RT. This methylation site is located on chr4:155661929 and cannot be annotated to any known genes to date. Some pieces of evidence support the conjecture that the DNA methylation of cg01511232 is associated with immune regulation and the risk of HIV infection [49]. Age is also considered to be a factor that causes the methylation alteration of cg01511232 [50]. These results suggested that cg01511232 is a risk signal for adverse health outcomes.

The CpG site cg08601457, which is mapped to the *FYN* gene and located on chr6:112115117, was found to be strongly relevant to our classification. The related pathways of *FYN* are RET signaling and adherens junction. FYN is an important molecular marker in breast cancer that can serve as a predictor of early recurrence [51]. The heterogeneous expression of FYN is also reported to have prognostic implications in lymphoma. FYN overexpression promotes cell proliferation and cell migration in various types of cancers and mediates epithelial–mesenchymal transition [52,53]. Therefore, the epigenetic modification at cg0601457 likely has a substantial influence on cancer progress that may be induced by the radiation effect.

A relatively high methylation level of cg23752651 indicated chest RT in the decision rules. This site is mapped to the *TNFRSF1A* gene, which is related to tumor necrosis factor-activated receptor activity. The specific methylation pattern at cg23752651 has been found in pancreatic ductal adenocarcinoma [54]. The R92Q variant in the *TNFRSF1A* gene reportedly influences susceptibility and phenotype depending on the age at disease [55]. Moreover, *TNFRSF1A* purportedly may serve as a diagnostic and prognostic biomarker in gliomas [56]. The polymorphism of *TNFRSF1A* is regarded as a predictive factor for RT-induced oral mucositis [57]. Therefore, we speculate that the methylation status of cg23752651 can indicate chest RT.

### 4.4. Key Methylation Alteration Related to Pelvic RT

The methylation site cg20112376 was found to be highly relevant to the classification for pelvic RT. This CpG site is located on chr4:6118443 and mapped to the *JAKMIP1* gene. An epigenome-wide association study found that cg20112376 is associated with long-term exposure to noise and air pollution [58], suggesting that the methylation status of cg20112376 represents a damaging burden of disease. The protein encoded by *JAKMIP1* plays a role in regulating microtubule cytoskeleton rearrangements. Expression changes in JAKMIP1 have been observed in the peripheral blood of patients undergoing RT [59]. In vitro experiments also demonstrated that radiation remarkably alters the expression of JAKMIP1 [60]. These findings supported the reliability of our analysis that cg20112376 can indicate pelvic RT.

The aforementioned CpG site cg21585138, which is mapped to the *CISH* gene, was also identified to be highly related to pelvic RT. It was found to have a role in indicating abdominal RT and associated with adverse health outcomes. This specific methylation pattern can be also caused by pelvic RT.

Another methylation site, cg21745092, was identified as a key feature. This CpG site is located on chr8:68868519 and mapped to the *PREX2* gene. The methylation level of cg21745092 is associated with age, suggesting a potential accumulation of lesions and a risk for disease [40]. *PREX2* can reportedly promote cell proliferation, invasion, and migration in pancreatic cancer [61]. *PREX2* plays an important role in regulating RAC activity and also participates in tumor susceptibility and disease progression [62]. We attributed the methylation status of cg21745092 to a certain environmental burden, such as radiation, that increases the risk for disease progression.

Among the decision rules for identifying pelvic RT, the hypermethylation of cg25531874 was found to be involved in many criteria. The CpG site cg25531874 is located on chr19:39440669 and mapped to the *FBXO17* gene. This gene is related to MHC-mediated antigen processing and presentation and innate immune response. Various associations between expression changes in FBXO17 and immune diseases have been reported [49,63]. The differential gene expression of FBXO17 has been found in numerous diseases, including breast cancer and gliomas [64,65]. These findings suggested that FBXO17 can be a biomarker for the risk of disease on the basis of the causal relationship between radiation and cancer progression. The methylation of cg25531874 can also serve as a potential biomarker for pelvic RT.

### 4.5. Limitations of This Study

This study also had some limitations. First, the feature selection methods: Boruta and mRMR were adopted to conduct this investigation. It was unknown whether they were optimum to process such methylation profiles. To date, lots of feature selection methods have been proposed. Additional essential methylation features, rules and better classifiers can be obtained with other feature selection methods. Second, several classification rules were extracted from each of the four RT datasets. However, we can only obtain elementary methylation patterns for patients who underwent different types of RT. Further deep investigations were still necessary. Finally, as a bioinformatics study, the new findings (methylation sites and rules) have not been validated by traditional experiments. We hope that related investigators can make further validations based on our findings.

## 5. Conclusions

In conclusion, our study computationally investigated the relationship between DNA methylation and RT. We first used the Boruta and mRMR methods to filter and rank features from four datasets, namely abdominal RT, brain RT, chest RT, and pelvic RT datasets. These feature lists were then sent into the IFS method, which used classification algorithms, such as DF, to find the best number of features and construct the optimal classifiers. Furthermore, decision rules for the quantitative description of the relationship between the methylation site and RT were developed. Several crucial methylation sites were identified to be highly associated with cancer RT, suggesting that RT has a substantial influence on DNA methylation patterns. We also revealed the specific methylation modifications associated with region-specific cancer RT, implying the different effects of radioactive exposures on specific body parts. These findings not only offer fresh insights into the regulatory role of methylation changes in cancer therapy but also provide a useful analytical approach.

## Figures and Tables

**Table 1:** Sample size of patients treated with different radiotherapy (RT).

Dataset	Positive Sample	Negative Sample	Total
Abdominal RT	412	1640	2052
Brain RT	629	1423	2052
Chest RT	577	1475	2052
Pelvic RT	352	1700	2052

**Table 2:** Detailed performance of different classifiers on four methylation datasets.

Dataset	Classifiers	Number of Features	Accuracy	Sensitivity	Specificity	MCC
Abdominal RT	DF	744	0.966	0.910	0.980	0.895
	kNN	10	0.846	0.971	0.814	0.662
	RF	753	0.903	0.913	0.900	0.739
	DT	761	0.791	0.825	0.783	0.515
Brain RT	DF	128	0.869	0.736	0.928	0.686
	kNN	8	0.749	0.863	0.699	0.519
	RF	115	0.811	0.765	0.832	0.577
	DT	150	0.690	0.693	0.688	0.355
Chest RT	DF	691	0.925	0.828	0.963	0.812
	kNN	12	0.804	0.945	0.749	0.627
	RF	234	0.851	0.823	0.862	0.654
	DT	489	0.762	0.747	0.768	0.478
Pelvic RT	DF	155	0.976	0.923	0.986	0.914
	kNN	9	0.841	0.977	0.813	0.637
	RF	31	0.896	0.906	0.894	0.702
	DT	77	0.798	0.795	0.798	0.487

**Table 3:** Total number of rules and the number of rules with each category generated by the optimal DT classifier in the four datasets.

Dataset	Number of Rules	Number of Rules for Positive Class	Number of Rules for Negative Class
Abdominal RT	151	87	64
Brain RT	239	132	107
Chest RT	166	93	73
Pelvic RT	183	99	84

## Data Availability

The data presented in this study are openly available at https://www.ncbi.nlm.nih.gov/geo/query/acc.cgi?acc=GSE169156 (accessed on 6 April 2021).

## Supplementary Materials

The following are available online at https://www.mdpi.com/article/10.3390/biology11040607/s1, Table S1: Feature lists were generated from the four methylation datasets (abdominal RT, brain RT, chest RT, and pelvic-RT) after Boruta feature selection and mRMR analysis; Table S2: Performance of different classifiers on the different numbers of methylation features on the abdominal RT dataset; Table S3: Performance of different classifiers on the different numbers of methylation features on the brain RT dataset; Table S4: Performance of different classifiers on the different numbers of methylation features on the chest RT dataset; Table S5: Performance of different classifiers on the different numbers of methylation features on the pelvic RT dataset; Table S6: Classification rules extracted by each of the best DT classifier from the different methylation datasets.
